# LC–MS-MS Determination of Cytostatic Drugs on Surfaces and in Urine to Assess Occupational Exposure

**DOI:** 10.1093/jat/bkac073

**Published:** 2022-09-15

**Authors:** José Ángel Lema-Atán, Elena Lendoiro, Lucía Paniagua-González, Angelines Cruz, Manuel López-Rivadulla, Ana de-Castro-Ríos

**Affiliations:** Toxicology Service, Institute of Forensic Sciences, University of Santiago de Compostela, Santiago de Compostela, Spain; Toxicology Service, Institute of Forensic Sciences, University of Santiago de Compostela, Santiago de Compostela, Spain; Toxicology Service, Institute of Forensic Sciences, University of Santiago de Compostela, Santiago de Compostela, Spain; Toxicology Service, Institute of Forensic Sciences, University of Santiago de Compostela, Santiago de Compostela, Spain; Toxicology Service, Institute of Forensic Sciences, University of Santiago de Compostela, Santiago de Compostela, Spain; Toxicology Service, Institute of Forensic Sciences, University of Santiago de Compostela, Santiago de Compostela, Spain

## Abstract

The ever-increased usage of cytostatic drugs leads to high risk of exposure among healthcare workers. Moreover, workers are exposed to multiple compounds throughout their lives, leading to cumulative and chronic exposure. Therefore, multianalyte methods are the most suitable for exposure assessment, which minimizes the risks from handling cytostatic drugs and ensures adequate contamination containment. This study describes the development and full validation of two liquid chromatography–tandem mass spectrometry methods for the detection of gemcitabine, dacarbazine, methotrexate, irinotecan, cyclophosphamide, doxorubicinol, doxorubicin, epirubicin, etoposide, vinorelbine, docetaxel and paclitaxel in working surfaces and urine samples. The urine method is the first to measure vinorelbine and doxorubicinol. For surfaces, limits of detection (LOD) and limits of quantification (LOQ) were 5–100 pg/cm^2^, and linearity was achieved up to 500 pg/cm^2^. Inaccuracy was between −11.0 and 8.4%. Intra-day, inter-day and total imprecision were <20%, except for etoposide and irinotecan (<22.1%). In urine, LOD and LOQ were 5–250 pg/mL, with a linear range up to 1,000–5,000 pg/mL. Inaccuracy was between −3.8 and 14.9%. Imprecision was <12.4%. Matrix effect was from −58.3 to 1,268.9% and from −66.7 to 1,636% in surface and urine samples, respectively, and extraction efficiency from 10.8 to 75% and 47.1 to 130.4%, respectively. All the analytes showed autosampler (6°C/72 h), freezer (–22°C/2 months) and freeze/thaw (three cycles) stability. The feasibility of the methods was demonstrated by analyzing real working surfaces and patients’ urine samples. Contamination with gemcitabine, irinotecan, cyclophosphamide, epirubicin and paclitaxel (5–4,641.9 pg/cm^2^) was found on biological safety cabinets and outpatients’ bathrooms. Analysis of urine from patients under chemotherapy identified the infused drugs at concentrations higher than the upper LOQ. These validated methods will allow a comprehensive evaluation of both environmental and biological contamination in hospital settings and healthcare workers.

## Introduction

Cancer is one of the most frequent diseases, with more than 19 million new cases diagnosed worldwide in 2020 (1). Cytostatic drugs are one of the several therapeutic strategies in cancer treatment. These drugs are highly toxic and cause adverse effects not only to patients but also to healthcare workers. The growth in the number of cancer patients together with the use of these drugs in non-malignant diseases (2) leads to the handling of ever-increased amounts of drugs. The toxic effects on workers have been known since the late 1970s, when Falck et al. (3) described the presence of compounds with mutagenic activity in the urine of exposed nurses. These effects include acute reactions, such as skin rashes, allergic reactions, headaches, dizziness, nausea, vomiting, diarrhea and hair loss, and long-term effects such as cancer, adverse reproductive outcomes (spontaneous abortion, congenital anomalies, fetal loss and infertility) and cytogenetic damage (chromosomal aberrations, micronuclei, sister-chromatid exchange and primary DNA damage) (4–7). Workers are exposed to multiple compounds throughout their lives, leading to a cumulative and chronic exposure. Therefore, multianalyte methods are the most suitable methods for exposure assessment, since methods with few drugs may underestimate the real risk of exposure (6, 8).

Different guidelines for cytostatic drug handling have been published by several organizations. The guideline published by the National Institute for Occupational Safety and Health (NIOSH) (9) is the most comprehensive one, providing recommendations, work protocols and scientific evidence of risk. However, no safe exposure limits have been established for cytostatic drugs due to a lack of consensus on the markers and analytical methodologies that should be used and the scarce scientific evidence on the correlation between exposure level and risk of adverse effects (10, 11). Despite the implementation of improved protection systems and security measures, there are still signs of exposure to cytostatic drugs (12–14), so the potential risk cannot be completely ruled out (4). Therefore, the recommended practice regarding exposure levels is following the ALARA principle (“as low as reasonably achievable”) (6, 7). Surveillance and control of exposure is another fundamental measure to help minimizing the risks arising from handling hazardous drugs and to ensure adequate containment of contamination. Moreover, it has been shown that routine monitoring campaigns have a positive effect on reducing contamination levels, serve to evaluate the effectiveness of the implemented prevention programs (10, 15, 16) and increase the workers’ concern and awareness (17, 18).

The present work aims to develop and validate two multianalyte methods for the simultaneous assessment of environmental and biological contamination, analyzing working surfaces and urine samples from healthcare workers involved in cytostatic drug handling. The selected drugs belong to nine structurally different families of compounds among the most used in the clinical practice.

## Materials and Methods

### Chemicals, reagents and materials

Cytostatic drugs (solid form) and deuterated internal standards (IStd) were purchased from Toronto Research Chemicals (Toronto, ON, Canada). LC–MS grade methanol (MeOH) was from Fisher Scientific (Hampton, NH, USA). Reagent grade formic acid (FA) (98–100%), HPLC grade dichloromethane (DCM) and 2-propanol (IPA) and LC–MS grade acetonitrile (ACN) were from Scharlau (Barcelona, Spain). Ethanol and sodium hydroxide were from Panreac (Barcelona, Spain). Ammonia (32%) was from VWR International (Radnor, PA, USA). Water was purified with a Mili-Q water system (Millipore, Le Mont-sur-Lausanne, Switzerland). VWR Light-Duty Tissue Wipers (11.4 × 21 cm) were from VWR International (Radnor, PA, USA). Solid phase extraction (SPE) cartridges Oasis^®^ HLB (3 cc, 60 mg) were from Waters Corporation (Milford, MA, USA) and MCX cartridges (3 cc, 60 mg) were from Teknokroma (Barcelona, Spain).

### Preparation of calibration curves and quality control samples for working surfaces

Individual stock solutions for each cytostatic drug and IStd were prepared at 1 mg/mL in MeOH from the solid forms. Then, a mixed stock solution containing all cytostatic drugs was prepared at 10 µg/mL using a mixture of 0.1% FA in water:ACN (90:10, v/v) as solvent (Solvent A). This solution was further diluted to obtain appropriate working solutions to generate the calibrators. Finally, an IStd-mixed stock solution was prepared in Solvent A at 10 µg/mL, which was further diluted to obtain a working solution at 1 µg/mL.

For the preparation of quality control (QC) solutions, two different stock solutions were made at 10 µg/mL. QC1 stock solution included dacarbazine, methotrexate, irinotecan, doxorubicinol, doxorubicine, epirubicine, vinorelbine, etoposide, docetaxel and paclitaxel, and QC2 stock solution included gemcitabine and cyclophosphamide. Both QC solutions were further diluted for the preparation of low, medium and high QC samples.

The preparation of calibrators and QC samples was made in a stainless-steel plate, which was divided into 18 segments of 10 cm × 10 cm. A 6- to 8-point calibration curve from 5 to 5,000 pg/cm^2^ was prepared by adding 250 µL of the corresponding working solution. QC samples were prepared by adding 250 µL of QC1 and QC2 working solutions to obtain samples at low (15 or 150 pg/cm^2^), medium (600 pg/cm^2^) and high (2,000 pg/cm^2^) concentrations. A blank sample was prepared with each calibration curve.

### Collection and extraction of working surface samples

Calibrators, QC samples and real working surface samples (100 cm^2^) were collected with VWR tissues previously moistened with 500 µL of Solvent A, wiping the selected area in three different directions (zigzag, vertical and horizontal) and folding the tissue after each sweep with the exposed side inward. After sampling, the tissue was placed in a polystyrene tube and, for real samples, stored at −22°C until extraction.

Before the analysis, 100 µL of IStd working solution and 4.5 mL of Solvent A were added to each tube. After horizontal shaking (15 min/400 rpm), the solvent was transferred through a POREX filter (POREX Corporation, Fairburn, GA, USA) into a glass tube and evaporated to dryness at 50°C. The dry extract was reconstituted in 100 µL of Solvent A, vortexed, transferred into a glass insert and centrifuged in a MiniSpin (10 min/14,500 rpm). Finally, the insert was placed into an injection vial, and 20 µL was injected into the HPLC–MS-MS.

### Preparation of calibration curves and QC samples for urine

From the individual stock solutions, two different mixed stock solutions for calibrators and QC were made at 10 µg/mL (Group A: dacarbazine, methotrexate, doxorubicinol, irinotecan, cyclophosphamide, doxorubicine, epirubicine, vinorelbine and paclitaxel) and at 50 µg/mL (Group B: gemcitabine and docetaxel), which were further diluted to obtain appropriate working solutions for the preparation of calibrators for Group A and 5 times more concentrated for Group B. The QC stock solutions were also diluted to obtain appropriate working solutions for the elaboration of low, medium and high QC samples. Finally, two different IStd stock solutions were prepared at 10 µg/mL for Groups A and B, which were diluted to prepare an IStd working solution at 2 and 10 ng/mL, respectively.

A blank urine pool (prepared with blank urine samples from laboratory personnel not in contact with antineoplastic drugs) was used for the preparation of calibrators and QC samples. A 6- to 8-point calibration curve from 10–250 to 1,000–5,000 pg/mL was generated by the addition of 50 µL of the appropriate working solution to 1 mL of blank urine samples. QC samples were prepared by adding 50 µL of the appropriate working QC solution to obtain samples at low (30, 150 or 750 pg/mL), medium (250 or 1,250 pg/mL) and high (750 or 3,750 pg/mL) concentrations. IStd working solution (50 µL) was added to achieve a final concentration of 100 or 500 pg/mL. A blank sample was prepared with each calibration curve.

### Collection and extraction of urine samples

Urine samples from patients under intravenous chemotherapy with some of the evaluated cytostatic drugs were collected to assess the feasibility of the method, for which they signed an informed consent. Specimen collection has the approval of the Galician Ethics Committee, Spain (Ref. Number 2018/587). Anonymized samples were frozen at −22°C until analysis.

Urine samples were extracted with two different SPE protocols, for which two aliquots of each sample (1 mL each) were conditioned with 2 mL of 10% FA in water. The first aliquot was extracted with Oasis^®^ HLB cartridges for the analysis of methotrexate, cyclophosphamide, docetaxel and paclitaxel. After cartridge conditioning with 2 mL of MeOH and 2 mL of water and sample loading, the cartridges were washed with 2 mL of water:MeOH (70:30, v/v) and then dried for 10 min. Finally, analytes were eluted with 3 mL of DCM:IPA (75:25, v/v). The second urine aliquot was extracted with MCX cartridges for the analysis of gemcitabine, dacarbazine, doxorubicinol, doxorubicin, epirubicin, irinotecan and vinorelbine. After conditioning the cartridge as previously described, the sample was loaded, and the cartridge was washed with 2 mL of 10% FA in water and 2 mL of MeOH. The cartridge was subsequently dried for 10 min and eluted with 3 mL of DCM:IPA:NH_4_OH (70:25:5, v/v/v). The eluates of both extraction protocols were separately evaporated to dryness at 37°C. Extracts were redissolved with 100 µL of Solvent A, vortexed, transferred into a glass insert and centrifuged in a MiniSpin (10 min/14,500 rpm). Finally, the insert was placed into an injection vial, and 10 µL was injected into the UPLC–MS-MS.

### LC–MS-MS

#### Working surface samples

An HPLC Alliance 2795 Separations Module with an Alliance Series Column heater/cooler coupled to a Quattro Micro^TM^ API ESCI^®^ triple quadrupole tandem mass spectrometer was employed (Waters Corp.). Chromatographic separation was performed using an Atlantis^®^ T3 column (100 × 2.1 mm, 3 µm) and maintained at 30°C. The composition of the mobile phase was 0.1% FA in water (A) and ACN (B) at a flow rate of 0.3 mL/min. The chromatographic gradient was as follows: 0–1 min 5% B, 1–6 min from 5 to 26% B, 6–9 min 26% B, 9–10 min from 26 to 40% B, 10–11 min from 40 to 70% B, 11–12 min from 70 to 100% B, 12–15 min 100% B, 15–15.5 min return to initial conditions and equilibrate for 5 min.

The MS was operated in electrospray in positive mode (ESI+), applying the following parameters: capillary voltage, 4.5 kV; source block temperature, 150°C; desolvation gas (N_2_) temperature, 400°C; desolvation and cone gas (N_2_) flow rates of 500 and 50 L/h, respectively. Detection was performed on multiple reaction monitoring (MRM) mode. MRM transitions, cone voltage and collision energy for each analyte (Supplementary Table S1) were automatically optimized by post-column infusion of each individual analyte (10 µg/mL). MassLynx 4.0 software was used to control liquid chromatography–tandem mass spectrometry (LC–MS-MS) system and data acquisition, and QuanLynx 4.1 was used for data processing.

#### Urine samples

An Acquity UPLC^®^ H-Class with a Quaternary Solvent Manager pump (Waters Corp.) coupled to a Xevo TQ-XS triple quadrupole mass spectrometer (Waters Corp.) was used. Chromatographic separation was performed using a CORTECS^®^ T3 column (100 × 2.1 mm, 1.6 µm), maintained at 30°C. The composition of the mobile phase was 0.1% FA in water (A) and ACN (B) at a flow rate of 0.3 mL/min. The chromatographic gradient was as follows: 0–1 min 5% B, 1–6 min from 5 to 26% B, 6–9 min 26% B, 9–10 min from 26 to 40% B, 10–11 min from 40 to 70% B, 11–12 min from 70 to 100% B, 12–12.1 min return to initial conditions and equilibrate until 15 min.

The MS was operated in ESI+ mode, applying the following parameters: capillary voltage, 1 kV; source block temperature, 150°C; desolvation gas (N_2_) temperature, 600°C; desolvation and cone gas (N_2_) flow rates of 1,000 and 300 L/h, respectively. MRM transitions, cone voltage and collision energy for each analyte (Supplementary Table S1) were automatically optimized by post-column infusion of the analytes (100 ng/mL). MassLynx 4.2 software was used to control the UPLC–MS-MS system and data acquisition, and TargetLynx XS was used for data processing.

#### Method validation

Validation of the methods for the analysis of working surfaces and urine samples was independently performed according to the Scientific Working Group for Forensic Toxicology (SWGTOX) recommendations. Evaluated parameters were linearity, limit of detection (LOD) and quantification (LOQ), inaccuracy, imprecision, interferences, matrix effect, extraction and process efficiency and stability.

Supplementary Table S2 shows the validation protocol and acceptance criteria for each parameter.

### Results and discussion

#### Method development and validation

Two different LC–MS-MS instruments and analytical methods were required for the analysis of surface and urine samples. An HPLC–Quattro Micro^TM^ API ESCI^®^ was used for the development and validation of surface samples as, according to the literature, sensitivity of this instrument would be enough for the analysis of real samples. However, expected urine concentrations in workers occupationally exposed to cytostatic drugs are much lower, for which the recently purchased UPLC–Xevo TQ-XS instrument was employed to achieve the low LOD required for urine samples. Analytical columns were similar, both silica-based, reversed-phase C18 columns. CORTECS^®^ T3 column had a lower particle size (1.6 µm) adapted to UPLC technology.

The developed methods allowed the identification of gemcitabine, dacarbazine, methotrexate, cyclophosphamide, doxorubicinol, doxorubicin, epirubicin, irinotecan, vinorelbine, docetaxel and paclitaxel in urine and also etoposide in working surfaces. Extraction of surface samples was performed by dilution with an acidic solvent. However, urine samples required extraction with two different SPE procedures (HLB and MCX cartridges) because a single procedure did not allow a proper sample cleaning and/or extraction of all the compounds. Etoposide could not be appropriately extracted with any of the two SPE methods and was excluded from the urine analysis.

Supplementary Figure S1 shows the chromatograms of the predominant MRM transition for each analyte in a VWR tissue wiper and in a urine sample fortified at the LOD.

There were some differences between the methods developed for the analysis of surface and urine samples regarding the IStd employed for the studied analytes and also the MS transitions monitored. In surface samples, the corresponding deuterated analog was used as IStd for each analyte, except for gemcitabine, for which dacarbazine-d_6_ was employed, and for anthracyclines (doxorubicinol, doxorubicin and epirubicin) and taxanes (docetaxel and paclitaxel), for which cyclophosphamide-d_4_ was employed. Paclitaxel-d_5_ was discarded because, unlike what was observed for paclitaxel, it produced a low and unsteady signal in the HPLC–MS-MS. For the other analytes, a deuterated analog was not commercially available when the method was developed. In urine samples, dacarbazine-d_6_ was also used as IStd for gemcitabine, and paclitaxel-d_5_ could be used for taxanes as the signal observed in the UPLC–MS-MS was stable. However, for anthracyclines, irinotecan-d_10_ was the selected IStd (all extracted with MCX cartridges) except cyclophosphamide-d_4_ (extracted with HLB cartridges). In addition, different MRM transitions were also selected for some compounds (gemcitabine, doxorubicin, epirubicin, vinorelbine, docetaxel and paclitaxel) for working surfaces and urine samples analysis, as the predominant fragment ions differed depending on the MS instrument used (Quattro Micro^TM^ or Xevo^®^ TQ-XS). For dacarbazine, the MRM transitions selected for the surface sample method produce interferences with the urine matrix in blank urine samples, and therefore, different fragment ions were monitored. Supplementary Table S1 shows the MRM methods employed for working surfaces and urine sample analysis.

The Xevo^®^ TQ-XS MS instrument incorporates a novel atmospheric pressure ionization source called UniSpray™ (US). This source is composed of a high-velocity heated nebulized spray directed toward a cylindrical, stainless-steel rod, called impactor, typically at 1 kV. The downstream gas flow hits the impactor off-center, following the curvature of the rod and heading toward the inlet orifice of the sampling cone. This is known as Coandă effect and promotes additional break-up and desolvation of droplets. The main difference between ESI and US is that in ESI the high voltage is applied to the spray capillary tip (2–5 kV), while in US, the high potential is applied to the impactor. However, US generates very similar spectra compared to ESI, producing predominantly protonated or deprotonated ions, but improving signal intensity (19). Nevertheless, this is an analyte and matrix-depending factor that must be evaluated for each application. In the present work, ESI and US were evaluated for the cytostatic drugs included in the methodology. US did not produce any signal for vinorelbine, docetaxel and paclitaxel. For the other compounds, ESI showed a higher signal intensity compared to US (ESI/US peak area ratio between 1.2- and 24-fold) for gemcitabine, doxorubicinol, doxorubicin, epirubicin and irinotecan, while the opposite happened for the rest of the compounds (ESI/US peak area ratio between 0.3- and 0.9-fold). On the other hand, ESI showed higher signal/noise ratio compared to US (ESI/US ratio between 1- and 5.7-fold) for those compounds with a greater signal intensity by ESI (gemcitabine, doxorubicinol, doxorubicin, epirubicin and irinotecan), while for dacarbazine, methotrexate and cyclophosphamide, the opposite occurred (ESI/US ratio between 0.1- and 0.9-fold), which indicated a better relationship among the peak area and the background noise. Nevertheless, ESI was the selected ionization source employed in the developed methods, as mentioned previously, due to the absence of signal for some compounds by US. To our knowledge, only one article has been published using US for the determination of 5-fluorouracil in wipe samples (20).

Method validation results for working surfaces and urine samples are shown in Table I. In working surfaces, paclitaxel, doxorubicinol, doxorubicin and epirubicin did not satisfy the acceptance criteria for linearity, inaccuracy and/or imprecision and, therefore, were only qualitatively validated. Similarly, doxorubicin and epirubicin were only qualitatively validated in urine.

**Table I.** Method Validation Results for Working Surfaces and Urine Samples

		Analyte											
Surface samples	Parameter	CP^a^	GEM^a^	DACA^a^	IRI^b^	MTX^b^	ETP^b^	DOCE^a^	VBN^b^	PACLI^c^	DOXOL^c^	DOXO^c^	EPI^c^
	Linearity (pg/cm^2^)	5–5,000	10–5,000	50–5,000	50–5,000	50–5,000	50–5,000	50–5,000	100–5,000	–	–	–	–
	LOD (pg/cm^2^)	5	5	5	5	5	50	50	100	50	50	100	100
	LOQ (pg/cm^2^)	5	10	50	50	50	50	100	100	–	–	–	–
	Inaccuracy (MRE)	–0.9 to 0.0	–2.0 to 8.4	–4.4 to 2.6	–10.3 to −7.5	5.3–6.5	–11.0 to 1.4	2.9–12.5	1.7–4.3	–	–	–	–
	Imprecision (%CV)	5.2–11.3	9.9–11.4	8.6–9.1	16.1–20.1	11.7–13.5	15.1–22.1	10.0–13.7	13.4–15.3	–	–	–	–
	Interference studies	No interferences											
	Matrix effect^d^	–58.3 to 1,268.9%											
	Extraction efficiency^d^	10.8–75%											
	Process efficiency^d^	15.5–683.9%											
	Autosampler stability	–4.2 to 4.5	–3.1 to 0.0	–1.9 to 2.4	2.9–3.2	–1.5 to −0.7	–1.1 to 1.1	–18.7 to −0.3	–3.0 to 8.6	–	–	–	–
	Freezer stability	–2.3 to −0.9	–12.0 to −4.4	0.3–2.0	0.2–1.8	–2.9 to −2.0	–0.4 to 0.0	–1.8 to 10.6	–9.5 to 6.6	–	–	–	–
		Analyte											
Urine samples	Parameter	CP^a^	GEM^a^	DACA^a^	IRI^a^	MTX^a^	DOCE^a^	VBN^a^	PACLI^a^	DOXO^c^	DOXOL^a^	EPI^c^	
	Linearity (pg/mL)	20–1,000	250–5,000	50–1,000	10–1,000	10–1,000	100–5,000	10–1,000	10–1,000	–	10–1,000	–	
	LOD (pg/mL)	20	250	50	5	5	100	10	5	50	10		10
	LOQ (pg/mL)	20	250	50	10	10	100	10	10	–	–	–	
	Inaccuracy (MRE)	–3.7 to −0.5	–3.8 to 0.7	2.4–4.4	–2.5 to 2.0	–4.1 to −3.4	–0.7 to 14.9	0.3–2.5	–3.6 to 0.3	–	–1.9 to 2.2	–	
	Imprecision (%CV)	4.3–5.9	11.2–12.4	3.9–5.1	3.7–6.2	5.5–9.6	2.9–9.5	3.7–8.3	2.6–7.1	–	6.6–7.5	–	
	Interference studies	No interferences											
	Matrix effect^d^	–66.7% to 1,636.0%											
	Extraction efficiency^d^	47.1–130.4%											
	Process efficiency^d^	28.5–1,438.8%											
	Autosampler stability	–0.8 to 3.1	8.9–9.5	–1.9 to 1.3	–4.0 to −2.8	2.4–2.7	–12.7 to −1.9	–11.3 to 2.0	0.2–2.1	–	–4.1 to 1.6	–	
	Freezer stability	–9.1 to −3.7	4.0–12.7	–10.2 to −8.6	1.5–7.2	–2.0 to 6.4	7.4–8.8	–3.1 to 12.2	–8.7 to −0.9	–	–0.5 to 4.4	–	
	Freeze/thaw stability	7.7–16.0	2.8–11.7	–19.8 to −19.0	–12.5 to −1.7	–6.0 to −5.8	–9.6 to 2.1	–0.2 to 6.0	–6.4 to −13.3	–	–13.1 to −11.3	–	

CP: cyclophosphamide, CV: coefficient of variation; DACA: dacarbazine; DOCE: docetaxel; DOXO: doxorubicin; DOXOL: doxorubicinol; EPI: epirubicin; ETP: etoposide; GEM: gemcitabine; IRI: irinotecan; MRE: mean relative error; MTX: methotrexate; PACLI: paclitaxel; VBN: vinorelbine.

a Linear regression and 1/*x* weighting factor;

b Quadratic regression and 1/*x* weighting factor;

c Qualitatively validated compounds;

d Specific ranges for each analyte are displayed in the Supplemental material (Tables S3 and S4).

Linearity was verified from 5–100 to 5,000 pg/cm^2^ and from 10–250 to 1,000–5,000 pg/mL in working surfaces and in urine, respectively (*r*^2^ > 0.99). For working surfaces, the SWGTOX acceptance criteria for residuals were not satisfied, except for gemcitabine, vinorelbine and cyclophosphamide. In this case, a more flexible criterion for residuals (within ±25% of the nominal value) was accepted, as previously used by other authors (17, 21), which was fulfilled in all cases. The reasons for accepting residuals within ±25% were 2-fold: (i) the difficulties for a homogeneous extraction of the drugs from the stainless-steel plate, where the sampling technique and the pressure exerted can play a role in the sampling efficiency, leading to a greater variability between the replicates of QC samples and calibrators; (ii) the purpose of the analytical method, which was the identification of trace levels of cytostatic drugs on working surfaces.

In working surfaces, acceptable results were achieved for inaccuracy, with calculated deviations of the nominal concentrations within −11.0 and 12.5%. Intra-assay, inter-assay and total imprecision were satisfactory, except for etoposide and irinotecan, for which %CV for total imprecision was below 22.1%. In urine, acceptable results were achieved for inaccuracy, with calculated deviations of the nominal concentrations within −4.1 and 14.9%. Intra-assay, inter-assay and total imprecision were satisfactory, with values of %CV below 12.4%.

No interferences were detected in blank samples fortified with the IStd or after fortification with different drugs of abuse and medicines. Endogenous interferences from tissue samples were not specifically evaluated since, unlike urine samples, all tissues have identical composition. No endogenous interferences were detected.

For working surfaces, matrix effect was not significant for irinotecan, cyclophosphamide and etoposide. Dacarbazine, gemcitabine and vinorelbine showed signal suppression, like their corresponding IStd. Signal enhancement was observed for methotrexate, doxorubicinol, doxorubicin, epirubicin, docetaxel and paclitaxel. For metotrexate, its deuterated analog was employed as IStd. For the other compounds, cyclophosphamide-d_4_ was used; however, reproducible enhancement results were observed (%CV = 8.6–17.6%). The high matrix effect in the group of taxanes was due to their poor signal in neat samples, so probably some components of the tissue were responsible for the signal enhancement. For urine samples, the matrix effect was not significant for methotrexate and docetaxel. Gemcitabine, dacarbazine, cyclophosphamide, irinotecan, doxorubicin, doxorubicinol and paclitaxel showed signal suppression, which was compensated in most cases by their deuterated analog. Only gemcitabine, doxorubicin and doxorubicinol did not use a deuterated analog as IStd. For doxorubicin and doxorubicinol, the suppression was similar to that of their IStd (irinotecan-d_10_). For gemcitabine, matrix suppression was lower than that of its IStd (dacarbazine-d_6_), but constant (CV < 20%); in addition, inaccuracy and imprecision criteria were met for this analyte. Finally, epirubicin and vinorelbine showed signal enhancement. For epirubicin, this allowed to obtain a lower LOD compared to doxorubicin. Parallel to surface samples, the signal obtained for vinorelbine was lower in neat samples than in samples fortified after extraction.

For working surfaces, extraction efficiency ranged between 10.8 and 75% and process efficiency between 15.5 and 683.9%. For urine, extraction efficiency ranged between 47.1 and 130.4% and process efficiency between 39.1 and 1,438.8%. Matrix effect, extraction and process efficiency results are summarized in Supplementary Tables S3 and S4.

All analytes were stable for 72 h in the autosampler at 6°C and after 2 months in the freezer at −22°C. In addition, all analytes were stable after three freeze-thaw cycles in urine samples.

#### Application to real samples

Twelve samples from working surfaces of the Pharmacy Service and Outpatients’ Area of the Oncology Unit of the Clinical University Hospital of Santiago de Compostela, Galicia (Spain) were collected to assess the applicability of the method. A 10 × 10 cm paper template was used to define the area to be wiped. The tested surfaces included biological safety cabinets (BSCs) and their surrounding floor, trolleys, countertops, trays, documentation desk, waste container lid and infusion pumps and their surrounding floor. In addition, the floor around the toilet in the outpatients’ bathrooms was also sampled. All the samples were taken after the working shift before cleaning, except for the outpatients’ toilet, which was randomly sampled during the shift, and the time of cleaning was not recorded.

Urine specimens from nine patients under treatment with some of the monitored cytostatic drugs were analyzed. These samples were only intended to verify the applicability of the method for the identification of the monitored compounds rather than quantification, since the analytical levels in the patients’ samples were expected to be much higher than the lower detection limits suspected in healthcare personnel occupationally exposed to cytostatic drugs.

Supplementary Table S5 shows the results in the positive real samples. In surface samples, contamination was detected inside two of the BSCs sampled. One BSC (contaminated with cyclophosphamide) had not been used for several days, which indicates an inefficient contamination removal. The other BSC (contaminated with irinotecan) had a plastic-backed disposable absorbent paper on the working surface, but contamination was also detected underneath. As expected, higher contamination with several cytostatic drugs (gemcitabine, irinotecan, cyclophosphamide, epirubicine and paclitaxel) was observed on the floor around the outpatients’ toilet, which was used by patients who come to the hospital to undergo treatment. In the urine samples, the prescribed cytostatic drugs were detected in all cases, and for Patient 8, a doxorubicin metabolite (doxorubicinol) was also detected.

#### Comparison to previously published methods

Most of the published methods for the analysis of cytostatic drugs in surface and urine samples included a limited number of compounds, many of them belonging to the same chemical family. Indeed, usually one to four analytes are monitored, and only few methods monitored a higher number of cytostatic drugs on surface (2, 5, 8, 16, 21–26) and in urine samples (4, 27). Moreover, in some of the published methods, different analytical instruments (GC–MS, GC--electron capture detector, LC–MS, LC–UV or LC--diode array detector) had to be employed for the analysis of the different drug families. The present manuscript allows the simultaneous analysis of 12 or 11 cytostatic drugs from nine or eight different chemical families in surface and urine samples, respectively, with LODs ranging from 5 to 100 pg/cm^2^ in surface samples and from 5 to 250 pg/mL in urine.

Sensitivity comparison among the published surface studies is somewhat difficult due to differences in the units of measure (ng/sample, pg/cm^2^ or ng/mL) and that the analytical methodology is not fully defined. In other cases, method detection limit values are given instead of LOD values, which also considers the recovery in the wiping and desorption steps (6–8), making the comparison even more difficult. In general, compared with other multianalyte procedures, higher sensitivity was achieved in the present manuscript, which is a key factor considering the low sample concentrations expected, especially in the urine of the healthcare workers occupationally exposed to the drugs. Specifically, lower LOD values were obtained for gemcitabine, dacarbazine, methotrexate, irinotecan, cyclophosphamide, vinorelbine and/or etoposide in working surfaces compared with other multianalyte methods wiping a 100 cm^2^ area (22–24). For the analytes described in the present work, Guichard et al. (21), who monitored 25 compounds, achieved LOD/LOQ values between 20 and 500 pg/cm^2^, being the only authors that also monitored vinorelbine, but with a LOD 5-fold higher than ours; and Dugheri et al. (2, 5), who included 21 cytostatic drugs, reported LODs from 0.25 to 2.95 pg/cm^2^, but they applied two different chromatographic methods to a sample area of 400 cm^2^. Similarly, other authors achieved lower LOD levels by sampling a surface area from 600 to 2,000 cm^2^ (8, 16, 25, 26).

With regard to the sensitivity of urine methods, only two methods monitored multiple cytostatic drugs in a single analysis. The method published by Fabrizi et al. (27) monitored 13 analytes and achieved higher LOD values than ours (30–670 pg/mL), except for methotrexate, for which the same LOD was reported (10 pg/mL); Izzo et al. (4) monitored seven compounds from five families, with LOD values between 2.5 and 5 pg/mL. Finally, the present work describes for the first time the analysis of vinorelbine and doxorubicinol (doxorubicine metabolite) in urine.

Regarding method validation, despite the numerous analytical methods developed to date in surface and urine samples, only few authors perform a complete method validation (25, 28). Linearity, accuracy, precision and LOD/LOQ are the most commonly validated parameters (6, 8, 20, 23, 29) in surface samples, although some authors also include the assessment of stability and recovery (21, 30, 31) or matrix effect (2, 5). On the contrary, other authors did not provide information about the validation carried out (32–38), or the information they provided was scarce (24, 39–42). Overall, unlike surface methodologies, the validation of the methods in urine was carried out in a more complete way, covering, in addition to parameters such as linearity, accuracy, precision and LOD/LOQ, others like recovery, stability, selectivity, matrix effect and carryover.

An important difference in the validation of the surface methods is the way to prepare the calibration curve. As mentioned previously, all calibrators and QC samples were spiked in a stainless-steel plate, and then, sampling was carried out. In contrast, in the previously published methods, calibrators were prepared by addition of the standard solutions to the sampling tissue in the collecting tube (2, 5, 20, 30, 43) or directly analyzing the standard solutions (21, 23, 44), omitting the sampling step. These procedures will result in an underestimation of the concentrations found in the real samples since they do not consider sampling and extraction efficiencies. In fact, we demonstrate a low sampling efficiency from the stainless-steel plate for several compounds (Supplementary Table S3). Thus, while recovery for the IStd directly added to the sampling tissue was nearly 100%, recovery for the non-deuterated analogs was 25–75%. Additionally, the use of urine calibration curves has been employed to quantitate wipe samples (37), which can also lead to an error in the real sample concentrations due to matrix effect.

In Spain, only González-Álvarez et al. (45) evaluated the surface contamination by gemcitabine, cyclophosphamide and 5-fluorouracil in three locations (BSC, validation table and administration table). Therefore, our methods will be the first in our region to allow a comprehensive evaluation of both environmental and biological contamination in hospital settings and in healthcare workers simultaneously to identify the risk factors and take the appropriate countermeasures to minimize the exposure.

## Conclusions

The present manuscript describes the development and validation of two LC–MS-MS multianalyte methods for the analysis of cytostatic drugs in working surfaces and urine samples, including a high number of analytes with similar or better sensitivity to that previously described in the literature. In addition, to our knowledge, this is the first time that vinorelbine and doxorubicinol are measured in urine samples. The methods were fully validated in both matrices. These methodologies are suitable to evaluate the level of contamination on environmental (working surfaces of oncology units) and biological samples (healthcare workers).

## Supplementary Material

bkac073_Supp
