# Supplementary material for: LC–MS-MS Determination of Cytostatic Drugs on Surfaces and in Urine to Assess Occupational Exposure
Source: J Anal Toxicol. 2022 Sep 15;46(9):e248–55. doi: 10.1093/jat/bkac073 (PMC9872221; doi:10.1093/jat/bkac073)
Supplement: bkac073_Supp [file bkac073_supp.zip › bkac073_Supp/SUPPLEMENTARY DATA.doc]

**SUPPLEMENTARY DATA**

**Table S1.** MRM transitions, retention time (R_T_), cone voltage (CV), collision energy (CE) and IStd for each compound for the analysis of working surfaces and urine samples.

|  | **WORKING SURFACES** | | | | | **URINE** | | | | |
| --- | --- | --- | --- | --- | --- | --- | --- | --- | --- | --- |
| **Compound** | **MRM Transition** | **R_T_ (min)** | **CV** | **CE** | **IStd** | **MRM Transition** | **R_T_ (min)** | **CV** | **CE** | **IStd** |
| Gemcitabine | 264.1>112  264.1>94.7 | 2.2 | 25  25 | 15  39 | Dacarbazine-d_6_ | 264.1>112  264.1>94.9 | 1.1 | 40  40 | 14  38 | Dacarbazine-d_6_ |
| Dacarbazine | 183.2>166.1  183.2>123.1 | 3.5 | 20  20 | 9  15 | Dacarbazine-d_6_ | 183.1>45.9  183.1>64.9 | 1.7 | 30  30 | 6  26 | Dacarbazine-d_6_ |
| Methotrexate | 455.3>308.5  455.3>134.1 | 7.2 | 30  30 | 31  35 | Methotrexate -d_3_ | 455,3>308.1  455.3>134.0 | 4.0 | 50  50 | 22  38 | Methotrexate-d_3_ |
| Irinotecan | 587.2>124.1  587.2>167.2 | 10.3 | 50  50 | 39  39 | Irinotecan-d_10_ | 587.3>124.1  587.3>167.1 | 7.1 | 80  80 | 34  36 | Irinotecan-d_10_ |
| Cyclophosphamide | 261.1>140.0  261.1>106.0 | 11.2 | 30  30 | 21  17 | Cyclophosphamide-d_4_ | 261.1>139.9  261.1>106.0 | 6.6 | 20  20 | 22  18 | Cyclophosphamide-d_4_ |
| Doxorubicinol | 546.3>399.5  546.3>363.5 | 9.8 | 20  20 | 13  25 | Cyclophosphamide-d_4_ | 546.2>399.1  546.2>363.1 | 6.6 | 30  30 | 12  24 | Irinotecan-d_10_ |
| Doxorubicin | 544.3>397.6  544.3>130.2 | 11.1 | 20  20 | 13  17 | Cyclophosphamide-d_4_ | 544.2>397.1  544.2>378.9 | 7.8 | 30  30 | 12  20 | Irinotecan-d_10_ |
| Epirubicin | 544.3>130.1  544.3>361.1 | 11.9 | 45  45 | 17  27 | Cyclophosphamide-d_4_ | 544.2>397.1  544.2>130.1 | 8.5 | 40  40 | 14  14 | Irinotecan-d_10_ |
| Etoposide | 589.2>229.2  589.2>185.1 | 13.1 | 20  20 | 41  21 | Etoposide-d_3_ | NA | NA | NA | NA | - |
| Vinorelbine | 779.6>122.1  779.6>357.2 | 13.2 | 60  60 | 50  49 | Vinorelbine-d_3_ | 779.4>122.1  779.4>658.4 | 10.3 | 60  60 | 48  24 | Vinorelbine-d_3_ |
| Docetaxel | 808.6>527.6  805.6>226.4 | 14.4 | 20  20 | 13  15 | Cyclophosphamide-d_4_ | 808.4>182.1  805.4>226 | 11.4 | 10  10 | 20  20 | Paclitaxel-d_5_ |
| Paclitaxel | 854.6>286.5  854.6>105.0 | 14.5 | 45  45 | 50  19 | Cyclophosphamide-d_4_ | 854.3>105  854.3>122 | 11.5 | 30  30 | 32  30 | Paclitaxel-d_5_ |
| Dacarbazine-d_6_ | 189.3>172.2 | 3.4 | 15 | 11 | - | 189.2>52.1 | 1.7 | 20 | 6 | - |
| Methotrexate -d_3_ | 458.3>311.6 | 7.2 | 30 | 19 | - | 458.2>311.1 | 4.0 | 50 | 20 | - |
| Irinotecan-d_10_ | 597.2>133.2 | 10.3 | 60 | 35 | - | 597.4>133.0 | 7.1 | 80 | 32 | - |
| Cyclophosphamide-d_4_ | 265.1>140.1 | 11.1 | 30 | 21 | - | 265.1>139.9 | 6.6 | 20 | 22 | - |
| Etoposide-d_3_ | 592,3>229.2 | 13.0 | 20 | 15 | - | NA | NA | NA | NA |  |
| Vinorelbine-d_3_ | 782.6>122.1 | 13.2 | 65 | 47 | - | 782.4>122.1 | 10.3 | 62 | 32 | - |
| Paclitaxel-d_5_ | NA | NA | NA | NA | - | 859.4>291.1 | 11.5 | 40 | 16 | - |
| NA: not analysed | | | | | | | | | | |

**Table S2.** Method validation procedure and acceptance criteria.

| **Parameter** | **Procedure** | **Criteria** |
| --- | --- | --- |
| Linearity | 5 calibration curves in 5 days. A zero sample (with IStd) was included each day | r^2^ ≥ 0.99  residuals ±20-25%* |
| LOD | 3 replicates in 3 days | Two MRM transitions  S/N > 3 and adequate IR** |
| LOQ | 3 replicates in 3 days | Two MRM transitions  MRE ±20%  %CV ≤20-25%* |
| Inaccuracy | 3 replicates at low, medium and high QC for 5 days | MRE ±20% |
| Imprecision | 3 replicates at low, medium and high QC for 5 days | %CV ≤20-25%* |
| Interference studies | IStd interferences: Zero samples with each calibration curve  Exogenous interferences: blank samples fortified with 48 compounds (and 23 additional compounds in urine) at 20 ng/cm^2^ (surfaces) and at 5 ng/mL (urine)  Endogenous interferences (only for urine): blank samples from 10 different donors fortified with the IStd. | No interferences |
| Matrix effect | At low and high QC by comparing average peak area of samples fortified after extraction with samples directly prepared in solvent A (n=4^†^; n=10^‡^) | - |
| Extraction efficiency | At low and high QC by comparing average peak area of samples fortified before extraction with samples fortified after extraction (n=4^†^; n= 5^‡^) | - |
| Process efficiency | At low and high QC by comparing average peak area of samples fortified before extraction with samples directly prepared in solvent A (n=4^†^; n=5^‡^) | - |
| Autosampler stability | At low and high QC by comparing processed samples stored for 72 hours at 6°C with fresh samples | Stability samples should quantify within ±20% of freshly prepared QC samples |
| Freezer stability | At low and high QC by comparing unprocessed samples stored for 2 months at -22°C with fresh samples | Stability samples should quantify within ±20% of freshly prepared QC samples |
| Freeze/Thaw (F/T) stability | At low and high QC by comparing samples after 3 F/T cycles with fresh samples | Stability samples should quantify within ±20% of freshly prepared QC samples |
| %CV: coefficient of variation, IR: ion ratio, IStd: internal standard, LOD: limit of detection, LOQ: limit of quantification, MRE: mean relative error, MRM: multiple reaction monitoring, QC: quality control, S/N: signal/noise, Solvent A: 0.1% formic acid in water:acetonitrile (90:10, v/v).  *The 20% criterion was applied to urine samples, while 25% was adopted for surface samples.  **Council Directive 96/23/EC.  ^†^Number of replicates for surfaces; ^‡^Number of replicates for urine samples. | | |

**Table S3.** Matrix effect (ME), extraction efficiency (EE) and process efficiency (PE) at low and high QC levels in working surface samples.

| **Compound** | **ME (%) (%CV) (n=4)** | | **EE (%) (%CV) (n=4)** | | **PE (%) (%CV) (n=4)** | |
| --- | --- | --- | --- | --- | --- | --- |
|  | **Low QC** | **High QC** | **Low QC** | **High QC** | **Low QC** | **High QC** |
| Gemcitabine | -51.6 (5.2) | -11.6 (5.0) | 71.4 (7.8) | 72.2 (2.2) | 34.6 (4.8) | 63.8 (2.8) |
| Dacarbazine | -29.1 (7.0) | -13.4 (1.4) | 53.9 (6.7) | 61.4 (8.7) | 38.2 (3.7) | 53.2 (2.9) |
| Methotrexate | 80.5 (7.6) | 76.6 (3.6) | 35.5 (9.9) | 43.0 (7.6) | 63.7 (11.0) | 76.0 (9.1) |
| Irinotecan | -6.3 (4.4) | -4.5 (7.3) | 52.7 (9.8) | 53.0 (12.4) | 49.4 (27.8) | 50.6 (8.6) |
| Doxorubicinol | 197.2 (9.0) | 174.8 (8.6) | 18.2 (18.2) | 24.8 (8.4) | 54.1 (32.9) | 68.3 (14.1) |
| Doxorubicin | 167.0 (11.8) | 204.2 (8.6) | 10.8 (27.3) | 15.2 (29.0) | 28.8 (64.2) | 46.3 (40.5) |
| Epirubicin | 224.7 (17.6) | 303.1 (14.2) | 12.1 (24.2) | 15.0 (8.5) | 39.4 (64.9) | 60.4 (38.0) |
| Cyclophosphamide | 1.6 (6.3) | 9.3 (3.1) | 74.3 (2.0) | 75.0 (5.9) | 75.5 (7.4) | 82.0 (5.1) |
| Etoposide | 1.9 (2.5) | -13.7 (6.6) | 60.1 (10.2) | 55.5 (21.6) | 61.3 (38.3) | 47.5 (11.9) |
| Vinorelbine | -38.6 (60.8) | -58.3 (49.7) | 25.2 (123.1) | 47.8 (124.9) | 15.5 (57.2) | 19.9 (54.5) |
| Docetaxel | 171.9 (10.4) | 18.6 (4.4) | 43.2 (29.0) | 47.4 (19.9) | 117.4 (93.7) | 56.3 (31.7) |
| Paclitaxel | 1268.9 (19.8) | 296.7 (33.3) | 50.0 (48.7) | 47.4 (57.8) | 683.9 (68.5) | 188.2 (57.2) |
| Dacarbazine-d_6_ | -27.7 (4.3) | -17.6 (3.3) | 98.9 (2.1) | 101.2 (6.5) | 71.5 (2.9) | 83.4 (3.6) |
| Methotrexate-d_3_ | 64.8 (4.9) | 66.3 (2.1) | 99.0 (3.7) | 92.1 (1.8) | 166.8 (10.2) | 153.2 (9.9) |
| Irinotecan-d_10_ | -3.9 (4.1) | -7.8 (5.4) | 103.1 (7.9) | 93.1 (14.0) | 99.1 (28.0) | 85.8 (7.9) |
| Cyclophosphamide-d_4_ | -1.5 (4.6) | 1.1 (3.3) | 101.8 (4.2) | 102.2 (7.1) | 100.2 (3.9) | 103.4 (5.2) |
| Etoposide-d_3_ | 2.1 (2.7) | -14.2 (9.1) | 96.3 (10.5) | 86.2 (18.6) | 98.3 (37.3) | 74.0 (13.5) |
| Vinorelbine-d_3_ | -45.8 (66.0) | -54.8 (46.9) | 119.0 (138.5) | 120.4 (131.3) | 64.6 (52.2) | 54.5 (58.6) |
| CV: coefficient of variation, QC: quality control | | | | | | |

**Table S4.** Matrix effect (ME), extraction efficiency (EE) and process efficiency (PE) at low and high QC levels in urine samples.

| **Compound** | **ME (%) (%CV) (n=10)** | | **EE (%) (%CV) (n=5)** | | **PE (%) (%CV) (n=5)** | |
| --- | --- | --- | --- | --- | --- | --- |
|  | **Low QC** | **High QC** | **Low QC** | **High QC** | **Low QC** | **High QC** |
| Gemcitabine | -33.0 (18.4) | -31.8 (18.1) | 62.8 (4.4) | 71.8 (3.0) | 41.8 (30.6) | 49.0 (5.8) |
| Dacarbazine | -66.7 (19.3) | -65.4 (19.6) | 94.4 (4.1) | 82.4 (2.1) | 31.5 (14.6) | 28.5 (8.1 |
| Methotrexate | 14.6 (27.0) | 15.9 (26.8) | 71.7 (4.5) | 72.2 (3.5) | 82.1 (7.5) | 83.7 (4.4) |
| Doxorubicinol | -9.1 (26.2) | -21.5 (29.2) | 59.3 (8.7) | 66.8 (2.8) | 53.9 (11.3) | 52.5 (3.3) |
| Irinotecan | -32.6 (22.5) | -33.2 (24.5) | 68.3 (6.3) | 80.7 (2.9) | 46.1 (12.5) | 55.3 (22.7) |
| Cyclophosphamide | -54.5 (13.9) | -64.4 (27.2) | 96.6 (17.3) | 88.4 (4.3) | 43.9 (2.3) | 31.5 (2.9) |
| Doxorubicin | -29.4 (34.8) | -16.8 (33.6) | 77.9 (14.3) | 47.1 (8.0) | 55.0 (12.4) | 39.1 (16.8) |
| Epirubicin | 24.4 (19.8) | 24.6 (16.1) | 49.2 (12.9) | 53.3 (9.6) | 61.2 (7.9) | 66.5 (17.5) |
| Vinorelbine | 394.2 (29.9) | 1636.0 (34.9) | 79.9 (3.4) | 82.9 (21.9) | 394.9 (93.5) | 1438.8 (83.2) |
| Docetaxel | -8.4 (15.2) | -2.4 (18.7) | 104.7 (18.2) | 109.9 (4.0) | 96.0 (19.9) | 107.4 (11.6) |
| Paclitaxel | -15.6 (21.0) | -25.5 (27.2) | 121.0 (6.6) | 130.4 (4.2) | 102.2 (5.1) | 97.1 (5.9) |
| Dacarbazine-d_6_ | -68.4 (18.6) | -69.2 (17.9) | 91.3 (6.0) | 86.3 (5.3) | 28.9 (11.1) | 26.6 (6.6) |
| Methotrexate-d_3_ | 11.7 (28.5) | 11.8 (28.8) | 72.6 (6.3) | 71.5 (4.7) | 81.1 (4.5) | 79.9 (3.6) |
| Irinotecan-d_10_ | -43.2 (26.3) | -38.2 (27.8) | 76.5 (3.6) | 88.9 (6.2) | 43.5 (13.5) | 54.9 (25.9) |
| Cyclophosphamide-d_4_ | -69.1 (31.0) | -69.1 (31.6) | 78.6 (2.0) | 84.8 (4.8) | 24.3 (4.0) | 26.2 (0.5) |
| Vinorelbine-d_3_ | 296.7 (29.2) | 1401.2 (33.4) | 100.3 (7.1) | 99.5 (21.6) | 397.8 (88.5) | 1493.6 (82.8) |
| Paclitaxel-d_5_ | -23.3 (14.2) | -26.6 (22.7) | 116.5 (9.7) | 127.6 (3.4) | 89.4 (6.0) | 93.7 (4.6) |
| CV: coefficient of variation, QC: quality control | | | | | | |

**Table S5.** Analytical results from working surface and urine samples.

| **Surface sample** | **Analyte/s detected (pg/cm^2^)** |
| --- | --- |
| Outpatient bathroom | Gemcitabine= 291.2, Irinotecan= 234.0, Cyclphosphamide= 24.1, Epirubicin= 10.5^a^, Paclitaxel= 4641.9^a^ |
| BSC-2 | Cyclophosphamide= 5.0 |
| BSC-3 | Irinotecan= 50.9 |
| **Urine samples** | **Analyte/s detected (>ULOQ^b^)** |
| Patient-1 | Irinotecan |
| Patient-2 | Irinotecan |
| Patient-3 | Docetaxel |
| Patient-4 | Paclitaxel, Gemcitabine |
| Patient-5 | Paclitaxel |
| Patient-6 | Cyclophosphamide, Epirubicin |
| Patient-7 | Cyclophosphamide |
| Patient-8 | Dacarbazine, Doxorubicin, Doxorubicinol |
| Patient-9 | Paclitaxel |
| BSC= biological safety cabinet  ^a^Qualitatively validated compounds. The numerical value is illustrative and is only intended to reflect the degree of exposure.  ^b^Concentrations found in urine specimens were above the upper limit of quantification (ULOQ) in all cases. | |

Figure S1. Chromatographic separation of cytostatic drugs on surface (S1A) and urine samples (S1B) fortified at the LOD.
